# The local mechanosensitive response of primary cardiac fibroblasts is influenced by the microenvironment mechanics

**DOI:** 10.1038/s41598-024-60685-4

**Published:** 2024-05-06

**Authors:** Nicoletta Braidotti, Giorgia Demontis, Martina Conti, Laura Andolfi, Catalin Dacian Ciubotaru, Orfeo Sbaizero, Dan Cojoc

**Affiliations:** 1 Department of Physics, University of Trieste, Via A. Valerio 2, 34127 Trieste, Italy; 2 CNR-Istituto Officina dei Materiali (IOM), SS 14 km 163.5, Area Science Park Basovizza, 34149 Trieste, Italy; 3 Department of Engineering and Architecture, University of Trieste, Via A. Valerio 6/A, 34127 Trieste, Italy; 4 Present Address: Department of Chemical and Pharmaceutical Sciences, University of Trieste, Via L. Giorgieri 1, 34127 Trieste, Italy

**Keywords:** Biophysics, Calcium signalling, Ion channel signalling, Applications of AFM

## Abstract

Cardiac fibroblasts (CFs) are essential for preserving myocardial integrity and function. They can detect variations in cardiac tissue stiffness using various cellular mechanosensors, including the Ca^2+^ permeable mechanosensitive channel Piezo1. Nevertheless, how CFs adapt the mechanosensitive response to stiffness changes remains unclear. In this work we adopted a multimodal approach, combining the local mechanical stimulation (from 10 pN to 350 nN) with variations of culture substrate stiffness. We found that primary rat CFs cultured on stiff (GPa) substrates showed a broad Piezo1 distribution in the cell with particular accumulation at the mitochondria membrane. CFs displayed a force-dependent behavior in both calcium uptake and channel activation probability, showing a threshold at 300 nN, which involves both cytosolic and mitochondrial Ca^2+^ mobilization. This trend decreases as the myofibroblast phenotype within the cell population increases, following a possible Piezo1 accumulation at focal adhesion sites. In contrast, the inhibition of fibroblasts to myofibroblasts transition with soft substrates (kPa) considerably reduces both mechanically- and chemically-induced Piezo1 activation and expression. Our findings shed light on how Piezo1 function and expression are regulated by the substrate stiffness and highlight its involvement in the environment-mediated modulation of CFs mechanosensitivity.

## Introduction

Cardiac fibroblasts (CFs) are non-excitable cells present in the heart in large amounts^1^. The key role of CFs consists of maintaining extracellular matrix (ECM) homeostasis, which, together with cardiomyocytes interaction, leads to the formation of a complex tissue texture^1,2^. This is subjected to substantial remodeling during most cardiac diseases where CFs homeostatic role is impaired. When subjected to stress or injury, CFs undergo an activation process that leads to a phenotypic conversion into myofibroblasts (MFs). MFs are characterized by a radically organized cytoskeleton and the neo-expression and incorporation of α-smooth muscle actin (α-SMA) into stress fibers. This ensures that the cells have the ability to exhibit higher contractility and thus higher traction forces^3,4^. Moreover, MFs increase the production of ECM proteins that induce tissue stiffening^5,6^, and result, therefore, in the primary drivers of fibrosis and related pathologies^1–4^. To investigate the features related to the phenotypic conversion of quiescent fibroblasts to MFs, several studies exploited the modulation of substrate stiffness showing that stiff environments are suitable for establishing MFs phenotype^2,7,8^. These findings suggest the existence of a positive feedback loop, in which MF-induced stiffening promotes the activation of new fibroblasts and further raises the stiffness.

Mechanosensitive ion channels^9,10^ are among the most important mechanosensors involved in signaling the mechanical changes of the microenvironment associated with the fibrotic condition^11^.

Several non-selective ion channels are expressed in cardiac fibroblasts, as reviewed in our previous work^12^. However, most of the Transient Receptor Potential (TRP) channels, some of which are also expressed by CFs and historically reported as elements involved in mechanosensory processes, have recently been confirmed to be intrinsically insensitive to membrane stretch^13^. Moreover, the Piezo1 channel^14^ has been demonstrated to work as a stretch-mediated upstream activator of some of these channels^15–17^ and has recently been confirmed as an important regulator of heart mechanobiology, able to initiate force-induced hypertrophic signaling in cardiomyocytes^18^.

Importantly, calcium has been suggested to sustain most of the myofibroblasts’ features by altering MFs collagen turnover, cell growth^19^, proliferation, and differentiation^20^. In addition, calcium signaling in CFs was proposed as a regulator of cardiac hypertrophy^21^, while mitochondrial calcium signaling was suggested as a regulatory mechanism in MFs differentiation and fibrosis^22^.

In light of this, we discussed in our previous work the evidence for Piezo1 involvement in the fibrotic mechanism and hypothesized the potential advantages of directly targeting Piezo1 in CFs in the context of cardiac fibrosis^12^. More recently, Piezo1 was confirmed to be an essential mechanosensor for cardiac MFs features, along with its overexpression in stiff environments^23^.

However, despite these promising accomplishments, a deep understanding of how various mechanical stimuli can regulate Ca^2+^ gating through mechanosensitive channels in CFs is still lacking. To quantitatively investigate this phenomenon, we mechanically stimulated primary CFs by local indentation and modulation of culture substrate stiffness, or a combination of both, and monitored their activity through calcium imaging. Local indentation was performed using two techniques: Optical Tweezers (OT) and Atomic Force Microscopy (AFM), which allowed us to span forces from piconewtons to nanonewtons and investigate a wide range of pressures, resembling those of the cardiac cycle. By culturing CFs on substrates with different stiffness (from GPa to kPa), we described how the mechanics of the environment modulates Piezo1 expression and activity in response to a local mechanical stimulus. We demonstrated that the Piezo1 channel plays a pivotal role in regulating the response of CFs across all physiological states (quiescent fibroblasts and myofibroblasts). Overall, our findings evidence the essential role of Piezo1 in controlling fibroblast’s mechanosensitivity, confirming its important role in microenvironment-mediated mechanotransduction, which is at the base of cardiac fibrosis.

## Results

### Local mechanical stimulation of primary rat CFs evokes force-dependent Ca^2+^ transients

We adopted a synergic approach combining OT and AFM to locally apply forces from few piconewtons to hundreds of nanonewtons and probe the CFs response.

**Figure 1:**
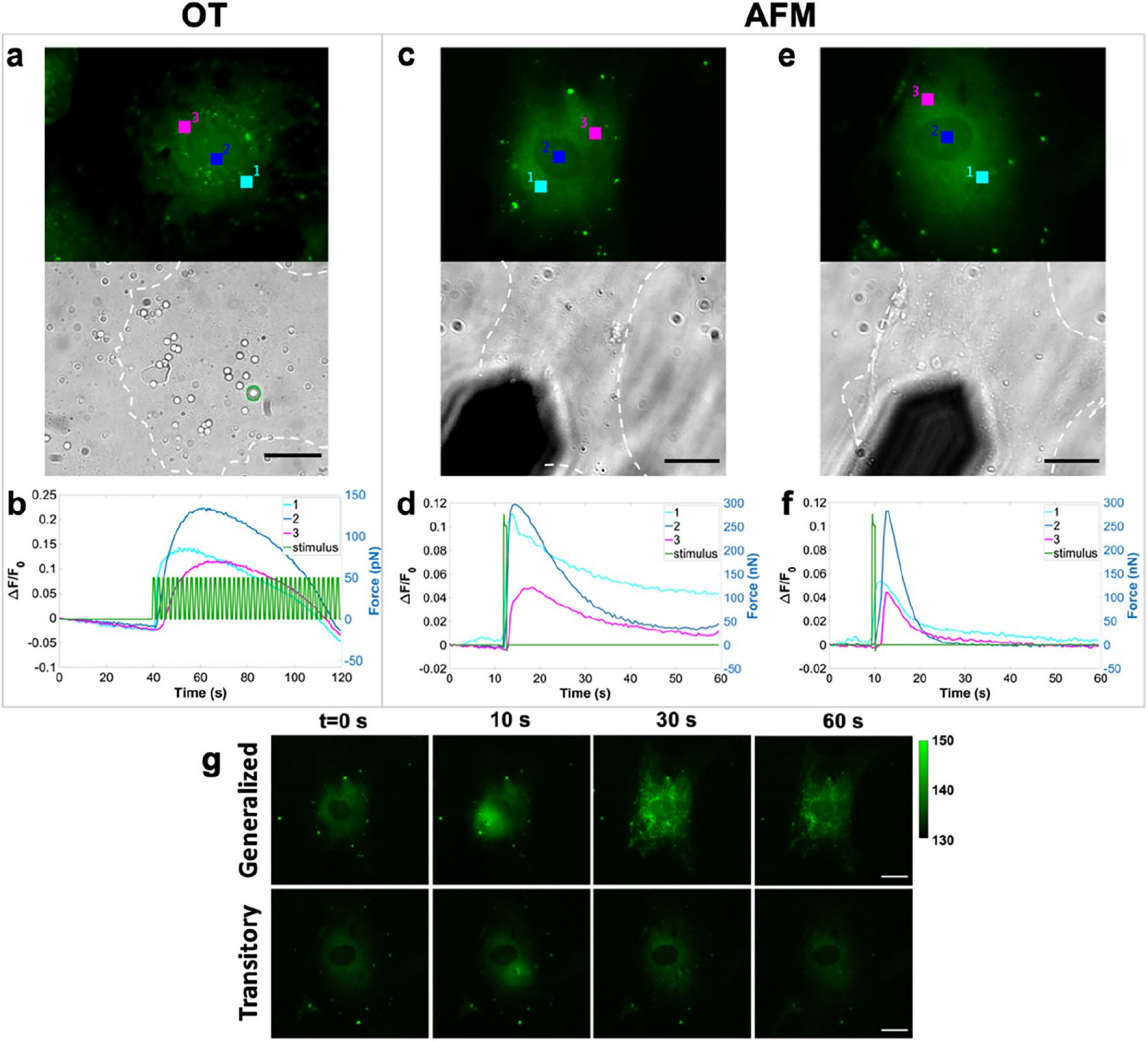
Stimulus-induced calcium signals are evoked by using OT (**a**, **b**) and AFM (**c**–**h**). In (**a**, **c**, **e**) the upper channel corresponds to the fluorescent signal while the lower one is relative to the bright field. Owing to the thin morphology of adherent fibroblasts which provides very low contrast in brightfield images, the cell boundary was marked for a better visualization. (**b**) The oscillating piconewton force applied by OT (green) is represented together with the calcium fluorescence ΔF/F0 signals. When the bead touches the cell membrane piconewton force pulses stimulate the cell and the fluorescence signal is detected first for ROI 1 (stimulated area, cyan signal) followed by its propagation to 2 (blue) and 3 (magenta). (**d**, **f**) Show examples of generalized and transitory responses respectively highlighting the differences in the decay times (τ). ROI 1 (cyan) represents the cell-tip contact point, and ROI 2 (blue) and 3 (magenta) are relative to areas in which calcium is propagated. Green traces correspond to the AFM stimulus. Scale bar: 20 μm. (**g**) Frames extracted from time-lapse movies at t = 0, 10, 30, and 60 s are reported for generalized and transitory responses. Calcium uptake in filamentous-like structures at the intracellular side is evidenced for the generalized response at 30 s. Even at 60 s some of these structures are still visible. Image contrast was adjusted to the same maximum and minimum intensity values and the corresponding color bar is reported on the top right. Scale bar 20 μm.

To exclude possible culture-specific artifacts, primary CFs from two different cultures were used, derived from different heart isolations. Cells were cultured for 24 h on stiff substrates (on the order of GPa) before the cellular response investigation. First, we used Optical Tweezers (OT) to apply piconewton forces of a maximum 50 pN^24^ in an oscillatory mode (0.5 Hz) to the cell membrane. Silica microbeads (3 μm diameter) were pipetted into the sample chamber and one bead was trapped above the cell (green circle indicated in the brightfield image of Fig. 1a). The trapped bead was moved up and down to touch the cell membrane exerting a maximum pressure of about 3 Pa after the bead came in contact with the cell membrane, which was evaluated by monitoring the force signal (green trace in Fig. 1b). An increment in the fluorescence signal can be detected when the trapped bead adheres to the cell membrane while the laser trap continues to move. This signal was first observed in the ROI 1 (fluorescence) near the bead-cell contact and then it is propagated towards the cell edge, as indicated by the signals corresponding to ROIs 2 and 3. At these pressures, the response of CFs was very weak, only 1% of cells responded, indicating that higher pressures are required to activate calcium-permeable mechanosensitive channels in CFs.

To increase the local pressure, we indented the cells with the AFM by using a microbead indenter (5 μm diameter) attached to the end of a tipless cantilever (Fig. 1c–f). At forces lower than 100 nN we did not observe any calcium signal. We then increased stepwise (50 nN) the applied force in the range of 150–350 nN (green trace in Fig. 1d, f) and a force-induced calcium response was detected. At these forces, by considering half area of the bead surface as the contact area^25^, the pressure applied ranges from 4 to 9 kPa, which is compatible with the physiological pressures reported for the cardiac cycle^26,27^.

By analyzing the fluorescence signals induced by the stimuli, we observed two distinct types of cellular responses, which we called “generalized” (Fig. 1d) and “transitory” (Fig. 1f). In the “generalized” response, the fluorescence signal occurs immediately after tip-cell contact and propagates across the whole cell. In this case, the fluorescence signal takes more than 10 s (τ > 10 s) to decay by a factor of 3 from maximum (Fig. 1d). Conversely, a faster decay (τ < 10 s) defines the “transitory” response (Fig. 1f).

In both cultures we observed an increment in the percentage of responsive cells as the applied force increases from 150 to 350 nN (Fig. S1a, b), with a slight shift towards higher percentages observed for the second culture with respect to the first one (Fig. S1a, b). The range of variability (see Table S1) is compatible with biological diversity that can be observed in primary cell cultures. These results confirm that a force-dependent behavior regulates Ca^2+^ gating and also the type of response. We indeed observed that the generalized response occurs preferentially at higher forces. In fact, the normalization of the mean generalized responses over the sum of generalized and transitory:

$$\frac{\% Generalized}{{\% Generalized + \% Transitory}}$$

shows an increment from 66% at 150 nN to 82% at 350 nN.

Interestingly, we also observed that, in cells responding with a generalized behavior, the initial cytosolic Ca^2+^ influx is followed by a long-lasting Ca^2+^ accumulation in inner filamentous elements that appear to be responsible for the longer decay time. To better understand this behavior, we analyzed the corresponding time-lapse fluorescence images. In Fig. 1g, frames extracted at time 0, 10, 30, and 60 s are shown for both the generalized (upper panel) and transitory (lower panel) responses. For the generalized response, 20 s after stimulus application, we notice the occurrence of Ca^2+^ accumulation in similar filamentous structures. Calcium release from these elements is relatively slow and, some of them are still calcium-enriched even 50 s after the stimulation. Such an event occurs preferentially at higher forces, where a higher percentage of generalized responses is observed, while it is less evident, or even absent, in transitory responses, as shown in the lower panel of Fig. 1g.

**Figure 2:**
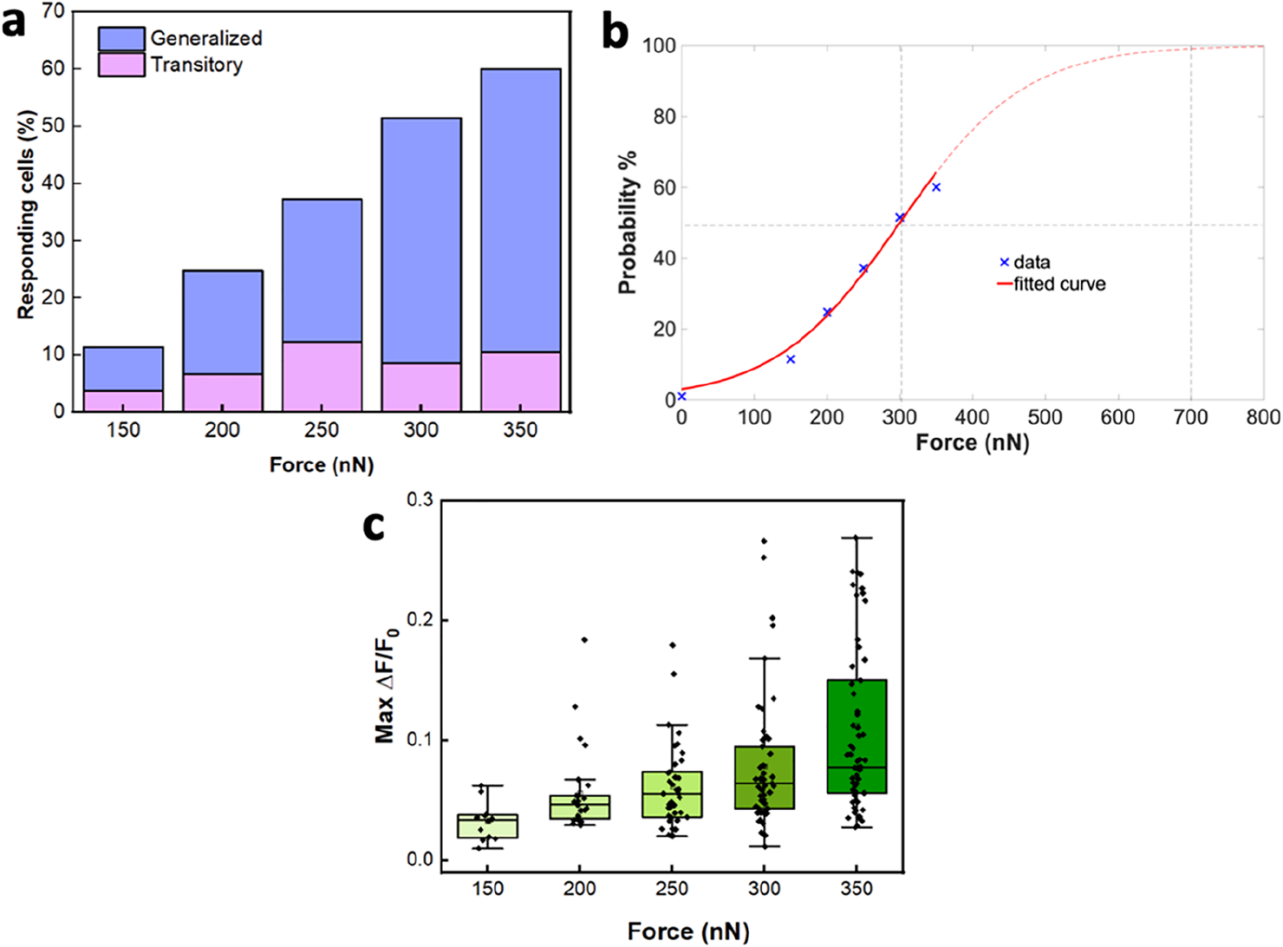
Channel activation probability and amount of mobilized calcium with increasing force. (**a**) Cumulative percentages of CFs that exhibit force-dependent behavior with generalized and transitory response. Cumulative percentages were evaluated considering all the responses coming from the two different cultures (N = 105 per each force). (**b**) Activation Probability as a function of force level. Experimental data (blue crosses) are fitted with the Boltzmann function (continuous red line) which can be extended to the higher forces (dotted red line). (**c**) ΔF/F0 distribution of responding cells as a function of the applied forces from 150 to 350 nN, (N = 150 per each force). Ca^2+^ mobilization follows a force-dependent behavior which suggests that at higher forces an increased number of channels is stimulated.

To better understand the relationship between the applied forces and the activation probability of mechanosensitive channels, we analyzed the cell response using the Boltzmann distribution^28,29^. The rationale for choosing the Boltzmann function is explained in detail in Supplementary Material (Comment S1). The analysis was performed on the cumulative results obtained from the two cultures (Fig. 2a), matching the trend observed for single culture (see supplementary Fig. S1). The cell can be considered as a system which can be in two states: non-activated and activated mechanosensitive channels, with a probability defined by the Boltzmann function, depending on the state’s energy. Part of the work done by the applied force (F) assists the transition from a non-activated to an activated state. Thus, the experimental data were fitted with the following function:

$$PA\left( F \right) = \frac{1}{{1 + exp\left[ { - \frac{{F - F_{0} }}{B}} \right]}}$$

F_0_ and B characterize the activation probability function. F_0_ is the force at which the activation probability is PA(F_0_) = 50% and it can be fixed to F_0_ = 300 nN on the base of experimental data: PA (300 nN) = 51.43%. With B as a free parameter, the fitting results were: B = 85.77 nN, with SSE = 0.0037 and R2 = 0.9857, indicating a very good fit (continuous red line in Fig. 2b).

Moreover, to estimate the calcium uptake as a function of the applied force, we evaluated the maximum ΔF/F_0_ values for each responding cell stimulated in the force range of 150–350 nN. We found that an increased amount of calcium was mobilized with increasing forces (Fig. 2c), supporting the hypothesis that with higher forces an increased number of mechanosensitive channels is stimulated. Background fluctuations were observed to be negligible, as demonstrated by the background fluorescence signals evaluation for each responding cell (Supplementary Material, Fig. S2).

The results presented in this section suggest that the force-dependent cell response probability can be well described by the Boltzmann function, which could be also used to predict the cell behavior at forces higher than those experimentally used in this work, and that the number of activated channels amplifies with the applied force, leading to an increased amount of calcium uptake.

### Piezo1 in CFs is localized also in mitochondria

**Figure 3:**
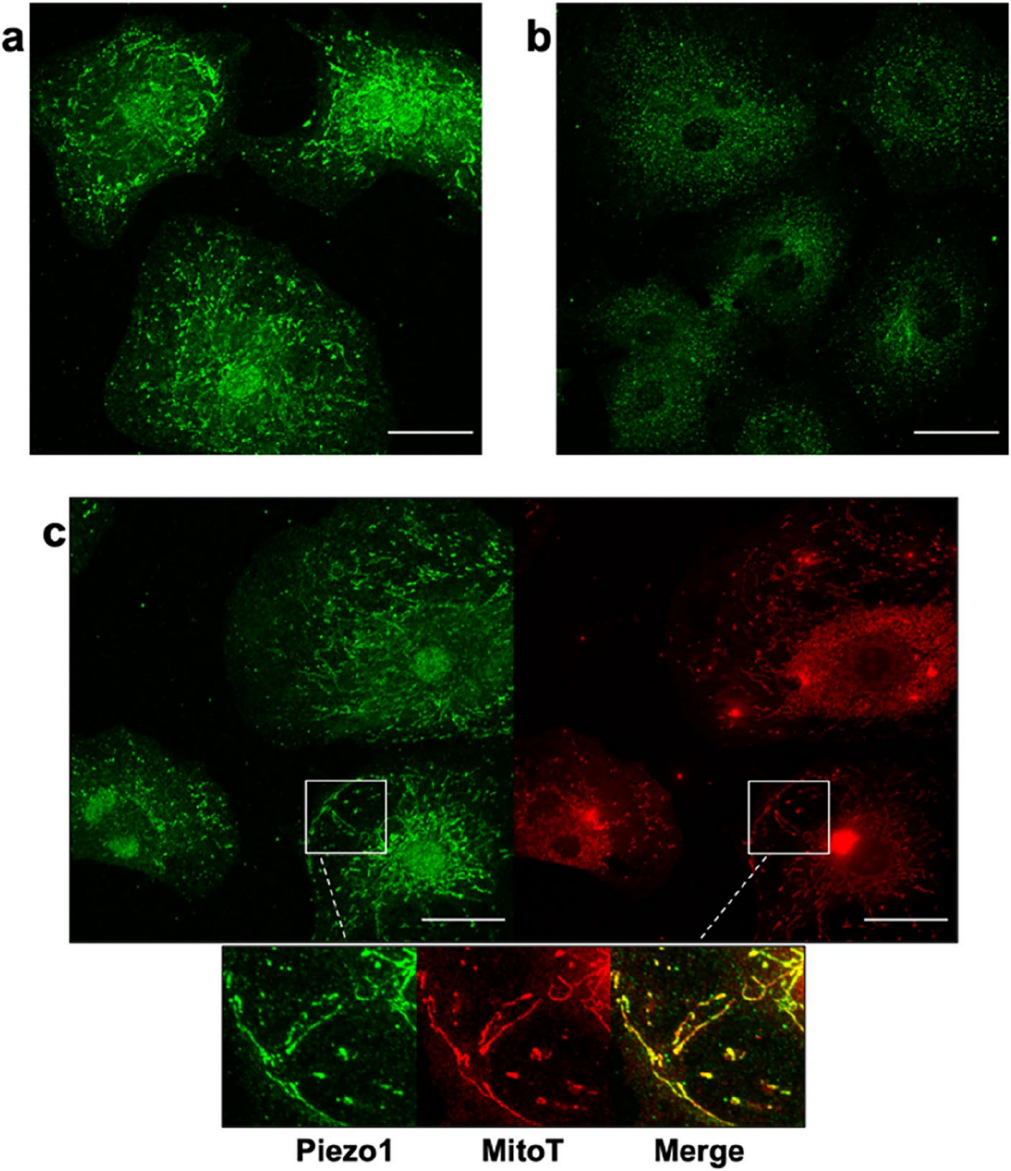
Piezo1 is colocalized with mitochondria. Permeabilized (**a**) and not permeabilized (**b**) samples stained for Piezo1 reveal a consistent protein expression both intracellular and at the plasma membrane. Permeabilized samples stained for both Piezo1 (green) and mitochondria (red) by using MitoTracker (MitoT) show the colocalization of signals (inlets) (**c**). Scale bar 40μm.

To understand the extent of Piezo1 involvement in the response of CFs to mechanical cues, we examined the presence and localization of Piezo1 in CFs by immunostaining and confocal imaging. The analysis was performed both on permeabilized (Fig. 3a) and not permeabilized (Fig. 3b) cells for accessing both outer and inner information on its localization.

We found that Piezo1 is consistently expressed in CFs and highly localized in intracellular filaments (Fig. 3a), with a distribution similar to that typical of mitochondria morphology^30,31^. To further investigate this observation, we double-stained permeabilized samples for Piezo1 and mitochondria. As demonstrated in Fig. 3c, the colocalization of mitochondria and Piezo1 was confirmed. To our knowledge, Piezo1 localization in CFs’ mitochondria is a new finding, previously observed only in pulmonary arterial smooth muscle cells^32^.

Interestingly, we noticed that this filamentous organization is also similar to the filamentous structures observed in the calcium response of cells stimulated at high forces (see Fig. 1g, upper panel). This would suggest that a micrometer pushing bead could also stimulate intracellular Piezo1 or even secondary messengers could be involved as a consequence of plasma membrane-mediated calcium influx inducing activation of intracellular channels.

### Myofibroblasts phenotype alters the mechanosensitive response to local stimulation

Based on the suggested Piezo1 involvement in cardiac fibroblast differentiation^12,23^, we focused our attention on the investigation of the mechanical response of fibroblast-activated phenotype.

**Figure 4:**
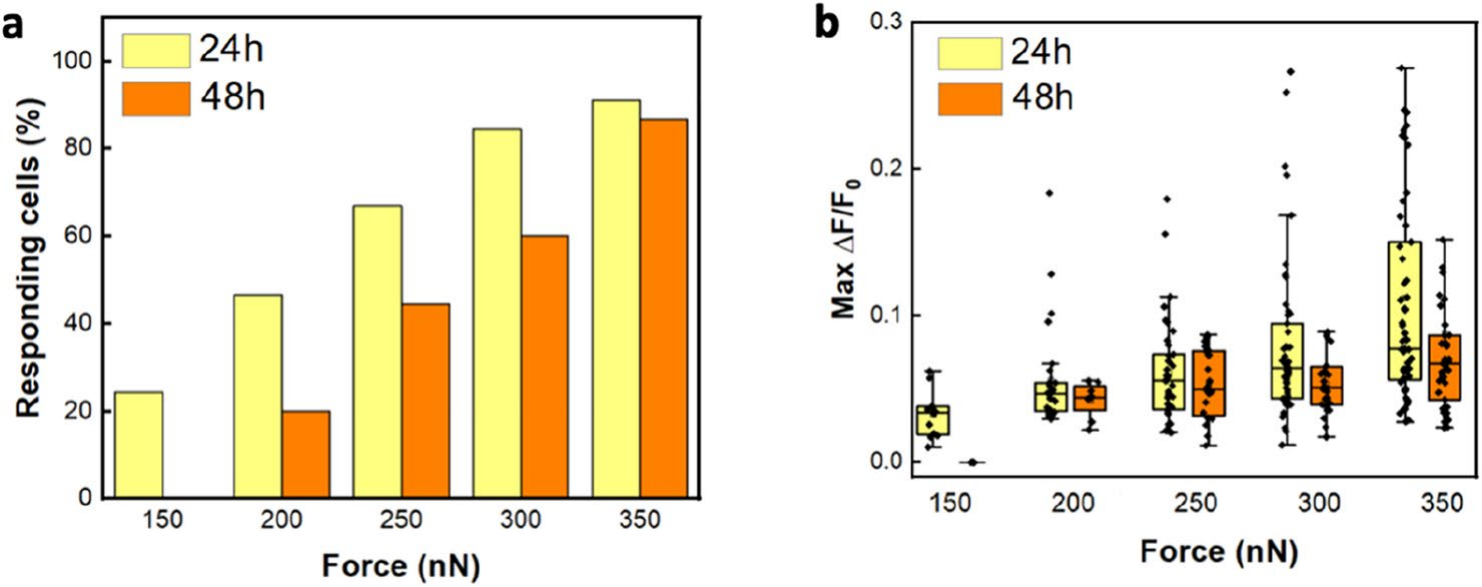
Differences in gating behavior between 24- and 48-h cultured cells (N = 45 per each force). The investigation of cells from the same culture and tested at 24 and 48 h highlights the reduction in channel activation probability (**a**) and in ΔF/F_0_ values (**b**) corresponding to lower calcium uptake and reduced stimulated channels number at 48 h.

A method to achieve a cellular population characterized by a naturally increasing amount of myofibroblasts is to extend the culturing time^33^. Therefore, we investigated the influence of a myofibroblasts-enriched population after 48 h of culture on the calcium gating response. We compared the results of mechanically stimulated cells at 24 and 48 h and observed that at 48 h the percentage of responding cells increases with force, but the increment is lower as respect to that observed at 24 h (Fig. 4a). To avoid artifacts due to culture-specific responses we investigated 24- and 48-h cultured cells (N = 45) coming from the same rat litter. In line with the observations made at the 24-h culturing time, we observed the occurrence of both transient and generalized responses in the cells after 48 h, also with a higher probability for generalized responses at higher forces (Supplementary Material, Fig. S3).

The analysis of the maximum fluorescence intensity (ΔF/F_0_) indicates a similar trend, with also negligible background fluctuations (Supplementary Material, Fig. S4), as for 24 h. However, by comparing the 24 h with the 48 h values, a reduction in ΔF/F_0_ at 48 h is noticed (Fig. 4b), which implies a lower calcium uptake.

Altogether these results indicate both a decrease in channel activation probability (Fig. 4a), and a decrease in channel number expressed at the stimulated area, as shown by a decrease in calcium signal at 48 h (Fig. 4b).

**Figure 5:**
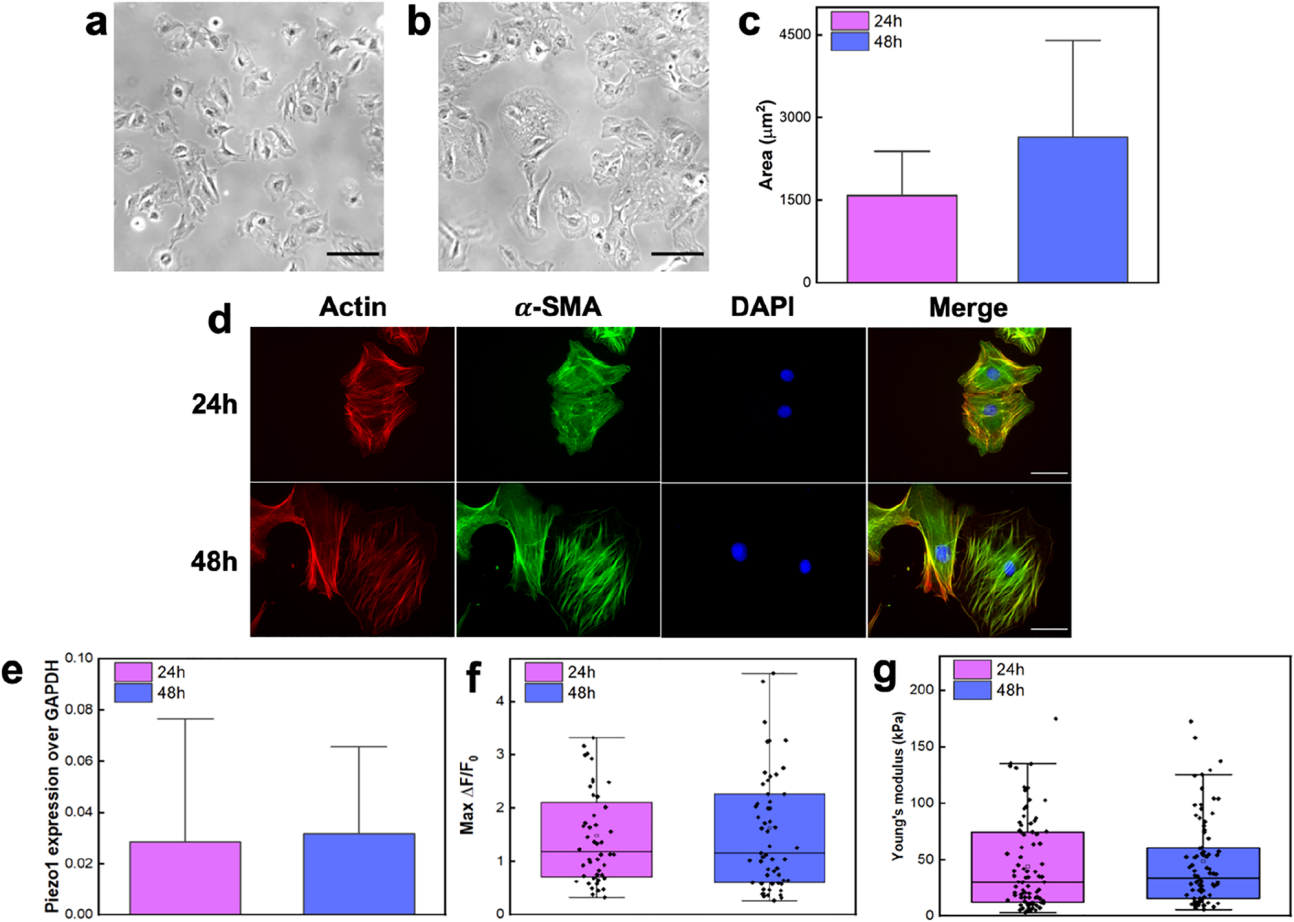
Morphological and mechanical investigations of cells together with Piezo1 expression for 24 or 48 h. Brightfield images (scale bar: 100 μm) for 24 h (**a**) and for 48 h of cultured cells (**b**) and surface area quantification (N = 61 for 24 h and N = 54 for 48 h) (**c**) show an increment in cell spreading with the time. (**d**) Fluorescence images for 24- and 48-h cultured cells were obtained by staining cells for actin (red), α-SMA (green), and DAPI (blue). At 48 h an increment in α-SMA incorporation into actin stress fibers is observed, as evidenced by the merged image. Scale bar: 40μm. (**e**) Piezo1 expression normalized to GAPDH for 24- and 48-h cultured cells (n = 3). (**f**) Distribution of ΔF/F0 values for all responding cells chemically stimulated with Yoda1 20μM (N = 49 for 24h and 60 for 48h). (**g**) Young’s Modulus measurement for 24- and 48-h cultured cells, N = 87. (**e**–**g**) demonstrate that neither changes in channel expression nor cellular mechanics are the underlying causes of the different gating behavior between 24 and 48 h.

To better investigate the reason for the different gating behavior and confirm the cardiac fibroblasts differentiation into myofibroblasts, we run brightfield and epifluorescence microscopy analyses of the samples at 24 h and 48 h. Compared to 24 h (Fig. 5a), at 48 h the cells show a larger cell spreading area (Fig. 5b). Such characteristic was quantified by approximating cellular shape to ellipsoid and then calculating its area. The result shows that at 48 h there is a significant increment in cell surface area with respect to 24 h (Fig. 5c). Moreover, this is also accompanied by increased actin stress fibers and α-SMA expression, mainly integrated with actin stress fibers rather than free in the cytoplasm (Fig. 5d), which also results in a higher percentage of α-SMA positive cells (Supplementary Material, Fig. S5). All these features support the fibroblast differentiation into myofibroblasts after 48 h on stiff substrates^2,33,34^.

Immunolabelling of transmembrane Piezo1 did not show substantial differences between 24 and 48 h culture (Supplementary Material, Fig. S6). Likewise, a similar channel expression between 24 and 48 h was confirmed by both qPCR (Fig. 5e) and by chemical stimulation with Yoda1, the selective agonist of Piezo1 (Fig. 5f). Although previous works supported the increased expression of Piezo1 channels in activated CFs^23,35^, we did not observe any relevant differences in protein expressions. This is most likely because the myofibroblasts phenotype is preferred over the quiescent one even at 24 h due to the stiff environment ^2^. The myofibroblast population increases over the time^33^ and relative variations in Piezo1 expression between 24 and 48 h could not be easily detected in the cell pool. On the contrary, those differences are more likely detected through mechanical stimulations performed on single cells.

In addition, we analyzed also the cell mechanics, to investigate whether the variation in cells elasticity could be responsible for a different gating mechanism. However, even in this case, we did not observe significant differences between the cell Young’s Modulus for the two culturing times (Fig. 5g).

These data suggest that the modulation of the gating behavior is not caused by changes in channel expression or cellular mechanics, but is rather regulated by the cell phenotype which is linked to a Piezo1 channel spatial reorganization. The increased spreading and a large integration of α-SMA into stress fibers characterize the cellular population at 48 h, which involves an increased amount of myofibroblasts, known to develop high traction forces and contractility^3,4^. Therefore, we can hypothesize that the different gating behavior of the Piezo1 channels may be correlated with these features. Indeed, Piezo1 was previously shown to relocate at focal adhesion sites during spreading^36^, and could therefore perhaps be hardly stimulated by vertical stimulation with a micrometer bead.

### Inhibition of myofibroblast phenotype reduces Piezo1 mechanical and chemical activation

To better understand the activity of Piezo1 channels in CFs phenotypes, cells were grown for 24 h on softer substrates, which can limit the activation of fibroblasts into myofibroblasts^2^.

PDMS produced in a ratio of 35:1, as described in Methods, refers to a stiffness of approximately 88 kPa^37–40^, a value that is about five orders of magnitude lower than that of common culture polystyrene Petri dish surface, which is typically on the order of GPa^41^.

**Figure 6:**
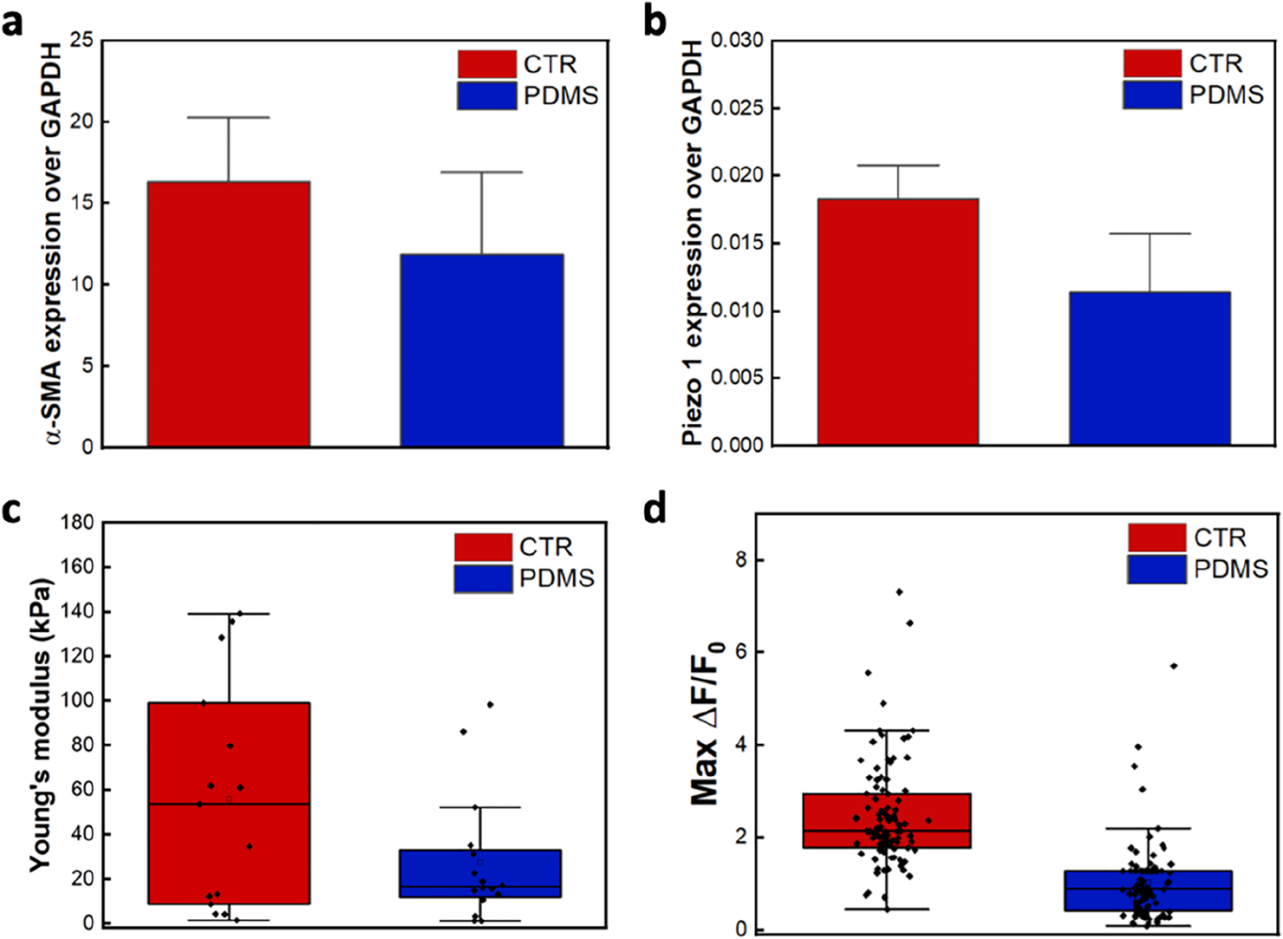
Inhibiting myofibroblasts content within the population lowered the cell elasticity and the channel expression, and, thus, also the mechanical and chemical channel activation. (**a**) α-SMA expression normalized to GAPDH for cells cultured on plastic (CTR) and elastomeric substrates (PDMS), n = 3. (**b**) Piezo1 expression normalized to GAPDH for cells cultured on plastic (CTR) and elastomeric substrates (PDMS), n = 3. (**c**) Young’s Modulus for cells cultured on plastic (CTR) and elastomeric surfaces (PDMS) (N = 17). (**d**) Distribution of ΔF/F_0_ values for all responding cells chemically stimulated with Yoda1 20μM (N = 93 for CTR and 76 for PDMS).

The analysis of α-SMA expression by qPCR confirmed a reduced fibroblast activation on softer substrates (Fig. 6a), in agreement with data reported in the literature^2,42^. At the previously determined threshold of 300 nN, no cell response was detected in cells cultured on PDMS with respect to the control, which instead confirmed a response of about 50%. This prompted us to formulate two hypotheses: (1) the channel expression could decrease; (2) the cell stiffness could decrease, requiring higher pressure to induce the membrane tension variation necessary to trigger the channel opening.

To assess these hypotheses, we performed several analyses. By using qPCR, we observed a decrease in Piezo1 expression on soft substrates (Fig. 6b), which is in agreement with the findings reported in ^23^. Analogously, the Young’s Modulus of cells decreases on soft PDMS substrates (Fig. 6c), in agreement with what is reported in^43^. Moreover, in this case, we observed a narrower data distribution than on rigid substrate, reflecting a homogeneous population of quiescent fibroblasts. In parallel, a significant difference in chemical activation of Piezo1 with Yoda1 was observed (see Fig. 6d), consistent with the reduced expression of the Piezo1 detected in qPCR analysis.

These results indicate that the probability of mechanically stimulating Piezo1 is reduced owing to a lower channel expression, and is likely to be further reduced by the cell softness, which could impair the proper conditions for Piezo1 activation, as also demonstrated in previous works^44,45^.

## Discussion

The alteration of mechanical stimuli from the cellular microenvironment is of great relevance in fibrotic diseases and determines the fate of the pathology. In particular, cardiac fibrotic remodeling, which is associated with almost every type of myocardial disorder, impairs cardiac compliance and relaxation, ultimately leading to heart failure^46,47^. The mechanisms that drive the feedback loop pathological remodeling in fibrosis are largely based on an impaired fibroblast mechanosensitivity^48,49^. Therefore, to better understand this pathological condition, it is relevant to further investigate the cellular mechanisms involved in sensing and transducing biomechanical cues. The CFs mechanosensitivity has been described by solely considering the investigation of the mechanical response of CFs to the modulation of substrate stiffness. Several works investigated how the mechanical microenvironment can regulate CFs phenoconversion^2,7,8^, cytoskeletal reorganization and proliferation^50,51^, and also calcium flickers^52^. To the best of our knowledge, no studies have been done on how local mechanical stimulation of CFs affects the function of mechanosensitive channels. This field is poorly investigated with fibroblasts of any origin, and few papers have been published up to date^53,54^.

In addition, it is important to consider that cells embedded in tissues are subjected to complex three-dimensional stimuli and, therefore, the mechanosensitivity of CFs can be more fully described by using a bimodal mechanical approach, that includes both local mechanical stimulation and substrates with different stiffness. As a result, here we investigated not only the local mechanosensitive response of fibroblasts but were also able to define how this response is modulated by the mechanical cues of the microenvironment. Thanks to this bimodal approach we found a force-dependent Ca^2+^ mobilization in CFs cultured on stiff substrates and observed that the calcium uptake involves both the cytoplasm and intracellular structures for higher forces. We observed that the CFs respond to the localized mechanical stimulation with either a “generalized” response, characterized by a slow decay, or a “transitory” one with a fast decay, as also observed in the case of cortical neurons^55^. Even if the physiological meaning of the two types of response is not clear, a dependence on the force magnitude exists, leading to a preferential occurrence of the generalized one at high forces. Therefore, we may suggest that high forces can promote the synergic activation of several channels localized on the plasma membrane, and/or at inner organelles of the cell. This is also confirmed by the increased amount of mobilized calcium at such forces. We may also think that the transitory response could trigger amplification mechanisms within the cell, leading to a more amplified generalized response. This could involve the activation of secondary messengers, recruitment of additional signaling molecules, or the modulation of cellular processes that extend beyond the initially stimulated region.

After 24 h of culture on stiff substrates, Piezo1 was found expressed all over the cell, with a strong localization at the level of mitochondria. Moreover, the intracellular filaments responsible for the long-lasting Ca^2+^ uptake, observed at high stimulating forces, were compatible with mitochondria organization. Such findings indicate that pushing the bead on the cell likely stimulates the whole cell, and that also Piezo1 located in intracellular regions might be activated at elevated forces both directly or as a consequence of secondary messengers. These results are in agreement with those obtained by Liao and coworkers^32^. They indeed demonstrated that, in pulmonary arterial smooth muscle cells, Piezo1 is expressed in intracellular organelles, including mitochondria, and responds to Yoda1 stimulation with intracellular calcium mobilization^32^.

Our results suggest that environment-mediated mechanical stimuli may induce a response in Ca^2+^ regulatory system, as mitochondria. Such organelles have been proposed to be involved in the fibrotic mechanism^22^. Indeed, the authors of^22^ demonstrated how some profibrotic factors affect fibroblast differentiation showing the involvement of mitochondrial Ca^2+^ regulation. Although mechanical profibrotic stimuli were not considered in this study, our results indicate that they could also be implicated in Ca^2+^ handling.

However, it remains to be precisely elucidated which is the role of Piezo1 at the mitochondria membrane and the pathway that regulates its possible activation in the force transduction process.

When the myofibroblast differentiation percentage was raised with 48 h-culture, we saw a decrease in cell responsiveness. Even in this case, we observed either a “generalized” or “transitory” response, with the generalized response exhibiting the same force-dependent trend. Since neither change in Piezo1 expression nor mechanics was detected, the different gating behavior was addressed to the change in cell phenotype which is likely linked to Piezo1 spatial reorganization. In fact, it is known that Piezo1 located on the plasma membrane undergoes reorganization during spreading by resulting largely recruited at the adhesion sites^36^. Moreover, the role of Piezo1 channel was proposed to be relevant in regulating traction forces, focal adhesion maturation, and substrate signaling^36,56,57^, all of which are critical characteristics of fibrotic-driven MF phenoconversion and persistence^58^. On the base of these findings, it is reasonable to think that Piezo1 results reorganized in 48-h culture cells, which could explain the reduced response of the cells in these conditions. In fact, Piezo1 channels located mainly at the adhesion sites and, thus, at the opposite side from our stimulation area, would be hardly activated. In addition, previous works demonstrated the existence of a link tethering Piezo1 and cytoskeleton, resulting in an alternative way for transducing forces ^59^. Therefore, it is likely possible that the different cell shape, actin, and α-SMA reorganization of CFs at 48 h could also contribute to modulating the gating behavior of Piezo1 on a stiff microenvironment.

On the contrary, when limiting MFs presence using soft substrates^2^, we found that Piezo1 expression decreases along with a sharp decrease in its chemical activation, which, in turn, results in a reduction of the mechanically activated calcium response. This behavior is in agreement with what was observed in ^23^, where the authors demonstrate that a soft cellular microenvironment determines a reduced Piezo1 expression in CFs.

Overall our findings suggest that Piezo1 is the main player in transducing mechanical cues in CFs, confirming the hypotheses made in our previous work^12^. This makes Piezo1 a suitable target for blocking mechanically-driven fibrotic mechanisms, in which it is most likely involved. Cardiac fibrosis and implicated heart functional impairments are still a debated big challenge as well as a serious health and clinical problem. Such cardiac remodeling driven by fibroblasts is a common feature of several cardiovascular diseases but effective specific therapies are still limited, and one of the main obstacles in this is the restricted knowledge about the fibrotic mechanisms^60–62^.

## Methods

### Cell culture

Post-natal day 6 neonatal Wistar rats were euthanized by decapitation (the study was approved by the OPBA Committee of the University of Trieste, under the Italian Ministry of Education, University, and Research, Protocol Code 1FF80.N.PZB. All experiments were performed in accordance with relevant guidelines and regulations.). The procedure for fibroblasts isolation was already shown in our previous work^63^. Briefly, hearts were extracted from the abdominal cavity and placed in CBFHH (calcium and bicarbonate‐free Hank’s Buffer with HEPES) supplemented with 10 U/mL heparin (H3149, Sigma‐Aldrich, St. Louis, MO, USA) and stored on ice until minced down to ~ 1 mm fragments. The tissue was then digested by trypsinization while the supernatant was being neutralized in calf serum and exchanged with fresh trypsin buffer every 5 min. Endothelial cells were discarded over the first two steps of such a trypsinization process. Cardiomyocytes and cardiac fibroblasts were sedimented at 300 RCF for 10 min, resuspended in fresh media (Dulbecco’s modified Eagle’s medium (DMEM) supplemented with 5% calf serum, 1% penicillin–streptomycin, 10 mg/L vitamin B12) and plated. After 1 h of plating the medium with unattached cardiomyocytes was removed and discarded. Thereafter, cardiac fibroblasts were cultured at 37 °C in a 95% H_2_O and 5% CO_2_ atmosphere in Dulbecco’s modified Eagle’s medium (DMEM), high glucose, GlutaMAX™, pyruvate (Thermo Fisher Scientific, Waltham, MA, USA), and supplemented with 10% fetal bovine serum (FBS) (Sigma‐Aldrich, St. Louis, MO, USA) and 1% antibiotic–antimycotic (Thermo Fisher Scientific, Waltham, Ma, USA). For all the experiments fibroblasts were used at P1 after about 5 days in culture before passaging. Cells at a density of 10.4 cells/cm^2^ were seeded in 35 mm Petri dishes and used after 24- or 48-h respect to the type of experiment. For experiments on Petri coated with PDMS, the surface of the elastomer was first activated under plasma oxygen and then incubated in 20 μg/mL fibronectin (F4759, Sigma‐Aldrich, St. Louis, MO, USA). In parallel, Petri dishes without elastomer were functionalized similarly and used as control.

For OT and immunofluorescence experiments, cells were plated on 18 mm diameter glasses at the same seeding density.

For qPCR, confluent cultures were cultured in T25 flasks or in petri dishes (60 mm diameter) prepared as described previously.

The study is reported in accordance with ARRIVE guidelines.

### Calcium loading protocol

Fluo-8 AM, green fluorescent calcium binding dye (ab142773, Abcam, Cambridge, England) was used at 4 μM. After PBS 1X wash, the fluorophore was incubated for 50 min at 37 °C in normal physiological saline solution (NPSS) composed by 140 mM NaCl, 5 mM KCl, 2 mM MgCl_2_,1 mM CaCl_2_,10 mM HEPES,10 mM Glucose. After incubation, the samples were washed two times with NPSS, and de-esterification in the same solution was performed at 37 °C for at least 15 min before the experiment. For Yoda1 experiment a solution of Yoda1 (CS-5095-5 mg, Chemscene, Monmouth Junction, NJ, USA) in NPSS was injected into the sample at a final concentration of 20 μM.

### OT stimulation and calcium imaging

The OT setup was used in several works of our lab previously^24^. For this work software improvement was done. The setup consists of an inverted microscope with a high numerical aperture objective (Olympus 60x, NA = 1.4 oil immersion, Olympus Corporation, Tokyo, Japan) and infrared laser at 1064 nm (IPG Laser, Burbach, Germany) which allows the optical trapping. A Focused Tunable Lens (EL-10-30- NIR-LD, Optotune AG, Dietikon, Switzerland) is computer-controlled by the software provided and adjusted to displace vertically the focus of the optical trap and move trapped 3 μm in diameter silica beads (Bangs Laboratories Inc, Fishers, IN, USA). For the mechanical stimulation, sinusoidal oscillations were performed at a frequency of 0.5 Hz and a laser power of 70 mW for the entire acquisition time. A fluorescence path is then coupled to the optical apparatus and consists of a fluorescence excitation source (X-Cite XLED1, Excelitas Technology, Waltham, MA, USA) which excites Fluo-8 AM at λ = 460 nm. The intensity of the LED was 25% with an external trigger set to 25 ms exposure with an interframe of 500 ms. Time-lapse images were then acquired with a CCD dual sensor camera (Orca-D2, Hamamatsu, Iwata, Japan) able to show bright field and fluorescent channels at the same time. The exposure of the camera was set to 25 ms and videos were acquired at a frame rate of 2 frames per second (FPS) starting about 10 s before performing stimulation. 100 cells coming from two different cultures, relative to different litters (one litter was made by 6 pups), were investigated.

### AFM stimulation and calcium imaging

For AFM-mechanical stimulation, silica beads of 5 μm in diameter were glued with UV-sensitive glue (NOA 73 Norland Optical Adhesive, Jamesburg, NJ, USA) at the end of tipless cantilevers (NANOSENSORS™, TL-FM-20, Neuchatel, Switzerland) with nominal spring constant (k) of 0.5–9 N/m. A silica bead was picked under microscopy control and cured for at least 30 min with a UV lamp. Before each measurement, the spring constant of the bead-mounted cantilever was calibrated in the fluid chamber of measurements by using the thermal noise method^64^.

The AFM module (NanoWizard II AFM JPK Instruments AG, Berlin, Germany) is coupled to an optical Axiovert 200 M microscope (Carl Zeiss AG, Oberkochen, Germany) equipped with an X‐cite® 120Q fluorescence illuminator (Excelitas Technologies Corp., Waltham, MA, USA), excitation-emission filter for FITC spectrum and a 40×/0.6 objective (LD Plan_Neofluar, Carl Zeiss AG, Oberkochen, Germany). Physical stimulations were performed as function of increasing applied forces: 150 nN, 200 nN, 250 nN, 300 nN, 350 nN while monitoring the fluorescence of calcium binding dye. The stimulation ramps consist in: (1) approaching the cell at 10 μm/s speed, (2) contact time 0.5 s, (3) cantilever retraction from the cell at the same speed. Each cell was stimulated only once.

Fluorescence time-lapse movies, started about 10 s before stimulation, for a total of 120 frames per stimulation at 2 FPS were recorded by an XM10 monochrome CCD camera (Olympus Corporation, Tokyo, Japan), connected to a 0.63× adaptor tube, at exposure time of 200 ms. Two different cultures, relative to different litters, have been investigated.

For chemical Piezo1 channel stimulation with Yoda1 a 20X/0.3 objective (LD A-Plan, Carl Zeiss AG, Oberkochen, Germany) was used. Time-lapse videos were recorded at 2 FPS for a total of 650 frames and started about 10 s before Yoda1 injection.

### Calcium transient analysis

The acquired fluorescence time-lapse movies were analyzed by a custom MATLAB code. In the case of mechanical stimulation (OT and AFM) a region of interest (ROI) was placed close to the physically stimulated cell area, and another one in the background. The differential fluorescence signal was calculated as follows:

$$\frac{\Delta F}{{F_{0} - B_{0} }} = \frac{{\left( {F - B} \right) - \left( {F_{0} - B_{0} } \right)}}{{F_{0} - B_{0} }}$$

where F is the fluorescence signal within the cell ROI, B is the fluorescence signal within the background ROI, F_0_ and B_0_ are the mean fluorescence signals calculated for the first n = 4 frames over the two ROIs.

In the case of mechanical stimulation by OT and AFM, the background fluorescence fluctuation was very low and hence neglectable (B = B_0_ with B_0_ ≈ 0). A cell was considered to respond if the stimulus induced a ΔF/F_0_ > 0.01.

For the analysis of Piezo1 channel chemical activation with Yoda1, fluctuations in the background (B) were observed due to the poor solubility of Yoda1 in aqueous media. In this case, the background signal could not be neglected and only responses exceeding the background fluorescence intensity variations (ΔF/F_0_ > 0.1) were considered.

### Evaluation of cell elasticity

Cell stiffness was obtained by evaluating the Young’s Modulus (E) of the cell, which was obtained by analyzing the force-distance curves acquired on single cells above the nuclei. Silicon nitride cantilevers (MLCT, E, Brucker, Camarillo, CA, USA) with a nominal spring constant of 0.1 N/m were used to indent the cell at 0.5 nN and 5 μm/s. The data analysis was performed by using the JPK DP software. To obtain the E value for each measured cell, the approaching part of the force-distance curve was converted into a force-indentation curve and fitted with the Hertz-Sneddon model (quadrilateral pyramid as indenter) at indentation depth of 200 nm, less than 10% of cell height^65,66^. Since our conditions satisfy the following requirements: δ ≤ 0.1 h and h ≥ 12.8R^67^, where δ = 200 nm (indentation depth), h≈2 μm (cell thickness) and R≈20 nm (probe radius of curvature), we could safely use the semi-finite sample assumption. On the contrary, corrections to the Hertz model would be required on thinner samples.

In addition, it is worth to noticing that some limitations exist in precisely evaluating cell elasticity on soft substrates, as highlighted by^68^. However, since they demonstrated that confounding errors coming from deformable substrates (0.1–20 kPa) are strongly reduced at 20–23 kPa stiffness, we can reasonably consider that in our case, due to the stiffer substrate we used, the deformation of the substrate can be neglected.

### Immunofluorescence

Immunofluorescence was performed for the observation of actin, α-SMA and Piezo1. Cells were seeded on round glass coverslips (18 mm diameter) for 24 or 48 h before being fixed in paraformaldehyde 4% for 20 min. In the case of permeabilized samples, cells were incubated for 5 min in 0.1% Triton X‐100. Both permeabilized and non-permeabilized samples were blocked with bovine serum albumin (BSA) 0.5% for 30 min. Thereafter cells were incubated for 1 h at room temperature in the following antibodies: primary antibody to mouse α‐SMA (ab7817, Abcam, Cambridge, England, 1:500) and rabbit Piezo1 (15939-1-AP, Proteintech, Rosemont, IL, USA, 1:150). Secondary antibodies used were: anti-mouse Alexa 488 (ab150113, Abcam, Cambridge, England, 1:500) and anti-rabbit IgG H&L (FITC) (ab6717, Abcam, Cambridge, England, 1:500). For actin staining phalloidin-iFluor 555 (ab176756, Abcam, Cambridge, England, 1:1000) was incubated with secondary antibody for 30 min at room temperature. Finally, DAPI (GTX16206, GeneTex, Irvine, CA, USA, 1:10) was incubated for 5 min. Samples were mounted by using ProLong™ Diamond Antifade Mountant (Thermo Fisher Scientific, Waltham, MA, USA, P36970). For mitochondria staining, living cells were incubated for 30 min at 1 μM with MitoTracker® Red CM-H2Xros (Thermo Fisher Scientific, Waltham, MA, USA, M7513) before being fixed.

Epifluorescent images were taken with microscope described in “AFM stimulation and calcium imaging” equipped with a 63X/1.4 Plan Apo oil immersion objective (Carl Zeiss AG, Oberkochen, Germany). Confocal images were acquired through a Nikon A1R confocal microscope (Nikon Instruments Inc., Melville, NY, USA) equipped with 403, 488, and 560 lasers, with a z step of 0.1 μm and a 60×/1.4 Nikon Plan Apo oil immersion objective (Nikon Instruments Inc., Melville, NY, USA).

α-SMA positive cells were evaluated by calculating the percentage as follows:

$$positive cells \left( \% \right) = \frac{Positive}{{Positive + Negative}}*100.$$

The percentage estimation was performed on two different cultures and by considering cells coming from at least 7 different sample areas for each time point and culture (n = 115 for 24 h and n = 70 for 48 h).

### Quantitative PCR

Total RNA from primary rat cardiac fibroblasts was extracted with Rneasy Protect Mini Kit (Quiagen, Hilden, Germany, 74,124). 1 μg of RNA was reverse—transcribed to cDNA using the PrimeScript RT Reagent Kit (Takara Bio USA Inc., San Jose, CA, USA, RR047A). Quantitative PCR was performed using SsoFast™ EvaGreen® Supermix (Bio-Rad, Hercules, CA, USA) on the CFSX384™ Real-Time PCR Detection System (Bio-Rad, Hercules, CA, USA). Plate was sealed and placed in CFSX384 Real-Time System (Bio-Rad, Hercules, CA, USA). GAPDH was used for normalization. Each sample in each group was detected in triplicate. The following sets of primers (Sigma‐Aldrich, St. Louis, MO, USA) were used in the study:

Piezo1 forward primer: TACTGGCTGTTGCTACCCTG;

Piezo1 reverse primer: CCTGTGTGACCTGGTATGCT;

α-SMA forward primer: CCTCTTCCAGCCATCTTTCAT;

α-SMA reverse primer: CGAGAGGACGTTGTTAGCATAG;

GAPDH forward primer: AAGTTCAACGGCACAGTCAAGG;

GAPDH reverse primer: CATACTCAGCACCAGCATCACC.

Resulting data were visualized with CFSX Manager software. Data are shown as 2^−(ΔCq)^ (mean ± STD). For comparisons of two groups, Student’s t-test was used.

### Fabrication of soft substrates

To fabricate soft culture substrates, polydimethylsiloxane (PDMS) was used and prepared by mixing the silicone elastomer with the crosslinker (Dow Corning Corporation, Midland, MI, USA, Sylgard™ 184 silicone elastomer kit) at a ratio of 35:1 to obtain a Young’s Modulus of 88 kPa^37–40^. After degassing in a vacuum chamber, the mixture was poured into a Petri dish (35 mm or 60 mm diameter) and cured in an oven at 65 °C for 15 h. Due to its intrinsic hydrophobicity, surface functionalization of the PDMS substrates is required to improve cell adhesion. For this purpose, after treatment with plasma O2 (40 W, 30 s), the substrates were incubated with fibronectin (20 µg/mL) for 30 min at RT and then washed in PBS 1X.

### Study approval

This study was approved by the OPBA Committee of the University of Trieste, under the Italian Ministry of Education, University, and Research (Protocol Code 1FF80.N.PZB).

### Supplementary Information

Supplementary Information.

## Data Availability

All data supporting the findings described in the manuscript are available in the article and in the supplementary information. Computer code: Any computer code used to produce figures, data acquisition and analysis is available upon request.
