# Supplementary material for: The local mechanosensitive response of primary cardiac fibroblasts is influenced by the microenvironment mechanics
Source: Sci Rep. 2024 May 6;14:10365. doi: 10.1038/s41598-024-60685-4 (PMC11074268; doi:10.1038/s41598-024-60685-4)
Supplement: Supplementary file 1 — Supplementary Information. [file 41598_2024_60685_MOESM1_ESM.pdf]

Supplementary Material

Supplementary Figures

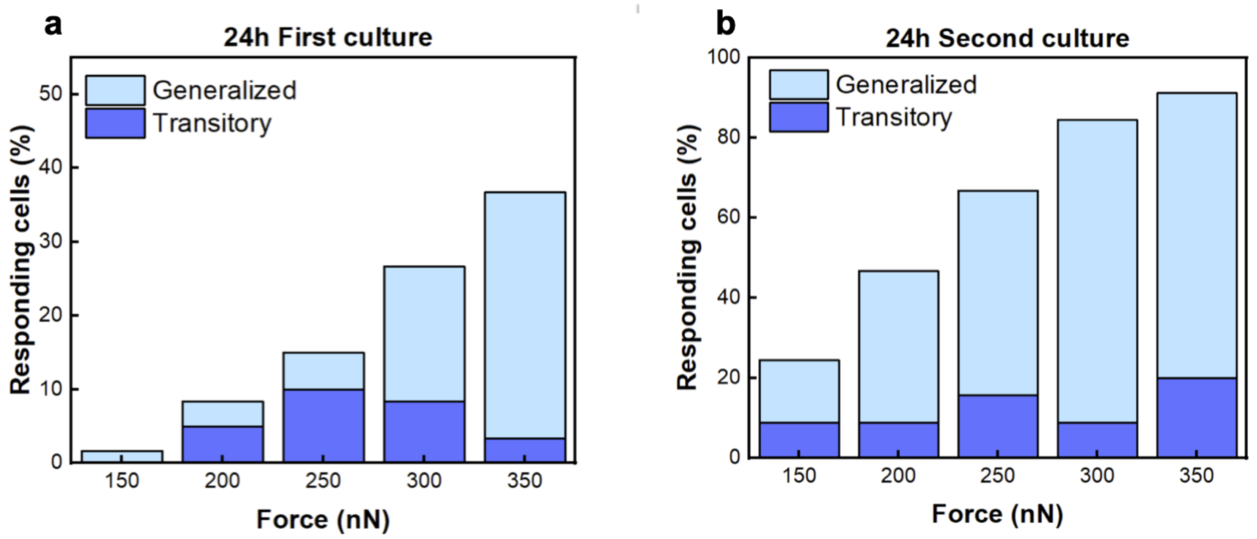

Figure S1. Force-dependent cell responses for the two distinct cultures. 60 cells were investigated for the first culture **(a)** and 45 cells for the second culture **(b)** per each force after 24 h plating. (See also the supplementary Table S1)

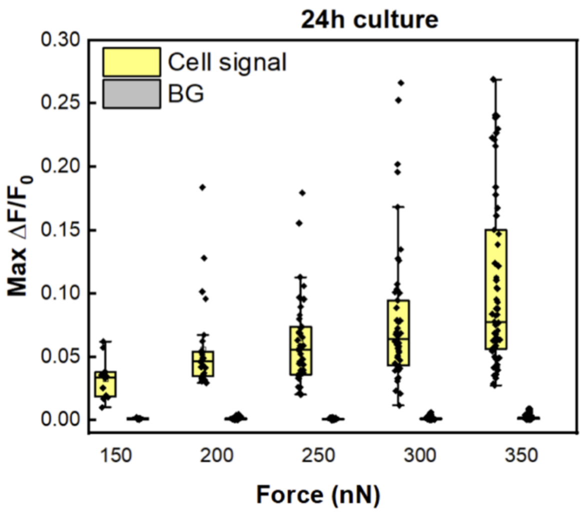

Figure S2. Distribution of $\Delta F/F_0$ values for all responding cells cultured for 24 hours and stimulated with forces from 150 nN to 350 nN, (N=105 per each force). BG refers to the $\Delta F/F_0$ values for each background in each responding cell and shows its negligibility respect to the cell response.

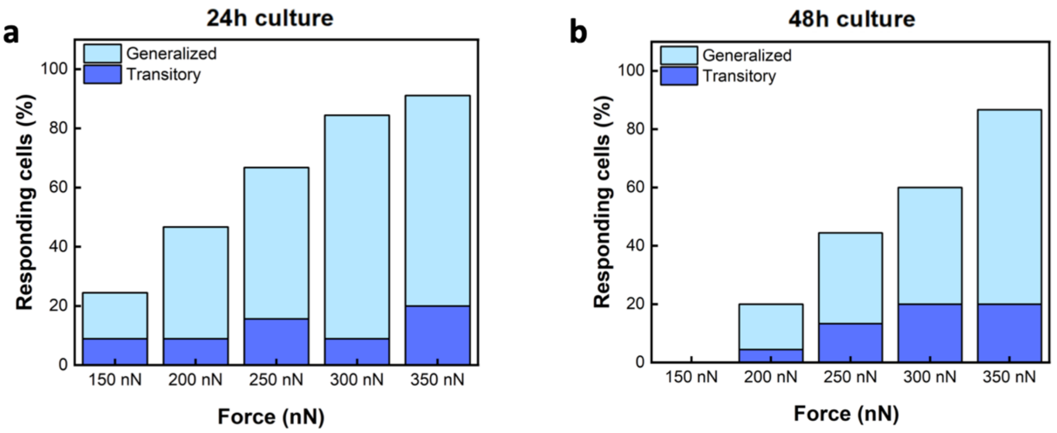

Figure S3. Percentages of CFs that exhibit force-dependent behavior with generalized and transitory responses to mechanical stimulation. Generalized and transitory responses are reported for 24 (**a**) and 48 h (**b**) cultured cells (N=45 per each force).

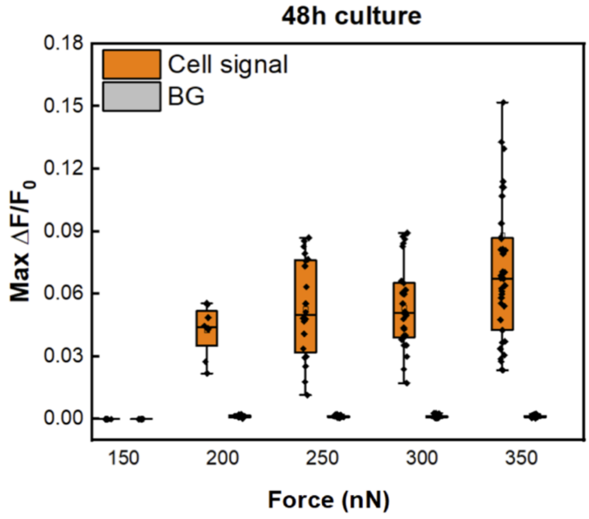

Figure S4. Distribution of $\Delta F/F_0$ values for all responding cells cultured for 48 hours and stimulated with forces from 150 nN to 350 nN, (N=45 per each force). BG refers to the $\Delta F/F_0$ values for each background in each responding cell and shows its negligibility respect to the cell response.

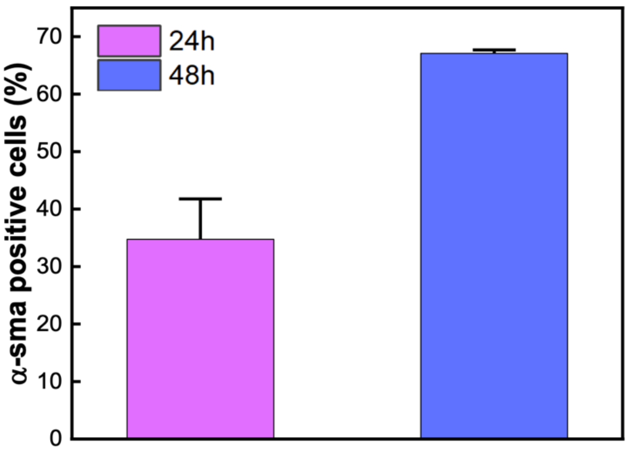

Figure S5. Evaluation of α-SMApositive cells from staining images performed on two different cultures and by considering cells coming from at least 7 different sample areas for each time point and culture (n=115 for 24h and n=70 for 48h).

a b

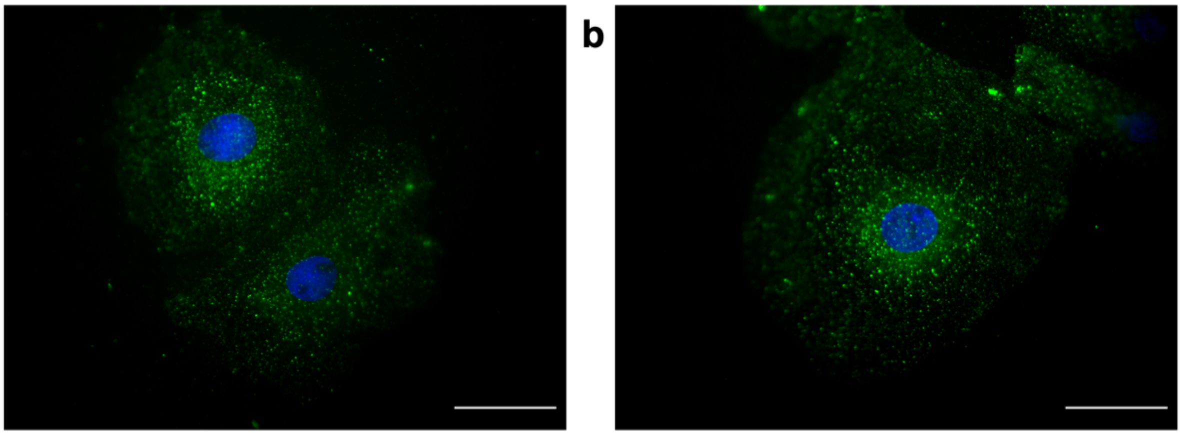

Figure S6. No detectable differences were observed in transmembrane Piezo1 between 24 **(a)** and 48 **(b)** hours cultured cells. Scale bar: 40μm.

Supplementary Table

First culture			
	Transitory %	Generalized %	Total %
150 nN	0	1.67	1.67
200 nN	5	3.33	8.33
250 nN	10	5	15
300 nN	8.33	18.33	26.67
350 nN	3.33	33.33	36.67
Second culture			
	Transitory %	Generalized %	Total %
150 nN	8.89	15.55	24.44
200 nN	8.89	37.78	46.67
250 nN	15.55	51.11	66.67
300 nN	8.89	75.55	84.44
350 nN	20	71.11	91.11
Mean ($\frac{\%_{first} + \%_{second}}{2}$)			
	Transitory %	Generalized %	Total %
150 nN	4.44	8.61	13.05
200 nN	6.94	20.55	27.50
250 nN	12.78	28.05	40.83
300 nN	8.61	46.94	55.55
350 nN	11.67	52.22	63.89
Range of variability ($\%_{max}$ - $\%_{min}$)			
	Transitory %	Generalized %	Total %
150 nN	8.89	13.89	22.78
200 nN	3.89	34.44	38.33
250 nN	5.55	46.11	51.67
300 nN	0.55	57.22	57.78
350 nN	16.67	37.78	54.44

Table S1. Percentages of responding cell for each type of response per culture. 60 cells were investigated in the first culture and 45 cells in the second culture per each force. Mean percentage values and ranges of variability are reported and highlight the data dispersion.

Supplementary Comments

Comment S1

Hereafter we explain the reason for which we adopted Boltzmann function for fitting our data (Figure 2b in the main text) and our interpretation.

As described in the manuscript, we performed mechanical stimulation experiments with the following experimental forces (Fex):

Fex= [0.1 150 200 250 300 350] in nN

The application of a mechanical force can trigger the transition of a cell at rest (non-activated) into an activated state, which corresponds to the opening of mechanosensitive channels (MsC), as indicated by the fluorescence signal.

For each force applied we probed N= 105 cells and found the following percentage of activated cells (PAex=activation probability):

PAex = [1; 11.43; 24.76; 37.14; 51.43; 60] %

The (Fex, PAex) data are plotted in the figure below (blue crosses).

To find a model that better represents the trend of our data, we considered the effect of the force at the equilibrium between the two cell states: activated and non-activated.

The probabilities corresponding to the two states are related by [Howard, J., Mechanics of Motor Proteins and the Cytoskeleton. 2001: Sinauer Associates]:

$$\frac{PA}{PN} = K_{eq} \exp\left[\frac{W(F)}{kT}\right] \quad (1)$$

$$PA + PN = 1 \quad (2)$$

where:

PN is the probability of the non-activated state;

PA is the probability of the activated state;

K_{eq} is the equilibrium constant of the spontaneous transition (from non-activated to activated) for the cells at rest (*F=0*);

W(F) is the work (energy) done to open the MsC, which is introduced by force application; *kT* is the thermal energy.

Considering the probabilities for spontaneous transitions equal: $PA_0=PN_0$, the equilibrium constant is $K_{eq}=1$.

The work W(F) is difficult to evaluate, but we know it is related to the force amount, which is probably converted into membrane tension to open the MsC.

However, to evaluate the relation between force and activation probability, we can rewrite eq (1) as:

$$\frac{PA}{PN} = exp\left[\frac{F-F_0}{B}\right] \qquad (3)$$

where F_0 represents the force for which PA= 50 %, and *B* is a free parameter with dimension of force.

From eq (2) and (3) we get the relation for the *PA(F)*:

$$PA(F) = \frac{1}{1+exp\left[-\frac{F-F_0}{B}\right]} \qquad (4)$$

From the experimental data, we observe that PA(300)=51.43 % is the closest value to 50 %. Therefore, we set F_0=300 nN, for which we get PA= 50 %, and then we fit the experimental data with the PA function described by eq (4), with B as a free parameter.

The experimental data (blue crosses) and the fit (red line) are plotted in the figure below:

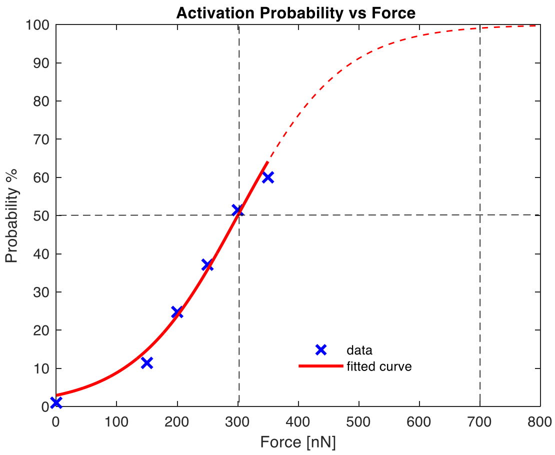

The free parameter B= 85.77, and the fitting function becomes:

$$PA(F) = \frac{1}{1+exp\left[-\frac{F-300}{85.77}\right]} \qquad (5)$$

with the following fitting error values:

- SSE – Sums of Squares due to Errors = 0.0037
- R_Square = 0.9857
- Root Mean Square Error RMSE = 0.0274

We wondered also which were the activation probabilities for higher forces. Since we could not implement stimulation experiments using forces F > 350 nN, we extrapolated the fit function to higher forces, as shown in the figure (red dotted line), and observed that, for a force F= 700 nN, the probability is already PA > 99 %.

In conclusion, the fitting analysis indicate that the experimental data can be properly fitted with a Boltzmann function, allowing to estimate the threshold force F_0=300 nN for which the activation probability is PA= 50 %.
